# Influence of ultra-low dose Aprotinin on thoracic surgical operations: a prospective randomized trial

**DOI:** 10.1186/1749-8090-3-14

**Published:** 2008-03-24

**Authors:** Efstratios Apostolakis, Nikolaos Panagopoulos, Efstratios N Koletsis, James Crockett, Helen Stamou-Kouki, Efrosini Sourgiadaki, Kriton Filos, Dimitrios Dougenis

**Affiliations:** 1 Department of Cardiothoracic Surgery, Patras University School of Medicine, Patras, Greece; 2 Department of Anesthesiology, Patras University School of Medicine, Patras, Greece

## Abstract

**Background:**

The blood saving effect of aprotinin has been well documented in cardiac surgery. In thoracic surgery, very few recent studies, using rather high doses of aprotinin, have shown a similar result. In a randomized prospective trial, we have tested the influence of aprotinin using an ultra-low dose drug regime.

**Methods:**

Fifty-nine patients, mean age 58 ± 13.25 years (mean ± SD) undergoing general thoracic procedures were randomized into placebo (Group A) and treatment group (Group B). The group B (n = 29) received 500.000 IU of aprotinin after induction to anesthesia and a repeat dose immediately after chest closure. A detailed protocol with several laboratory parameters was recorded. Patients were transfused when perioperative Ht was less than 26%.

**Results:**

The two groups were similar in terms of age, gender, diagnosis, pathology, co-morbidity and operations performed. The mean drainage of the first and second postoperative day in group B was significantly reduced (412.6 ± 199.2 vs. 764.3 ± 213.9 ml, p < 0.000, and 248.3 ± 178.5 vs. 455.0 ± 274.6, p < 0.001). Similarly, the need for fresh frozen plasma transfusion was lower in group B, p < 0.035. Both the operation time and the hospital stay were also less for group B but without reaching statistical significance (84.6 ± 35.2 vs 101.2 ± 52.45 min. and 5.8 ± 1.6 vs 7.2 ± 3.6 days respectively, p < 0.064). The overall transfusion rate did not differ significantly. No side effects of aprotinin were noted.

**Conclusion:**

The perioperative ultra-low dose aprotinin administration was associated with a reduction of total blood losses and blood product requirements. We therefore consider the use of aprotinin safe and effective in major thoracic surgery.

## Background

The incidence of postoperative hemorrhage after cardiopulmonary bypass is about 5–7% and after pulmonary resections is somewhat lower, about 4.6% [1,2]. Aprotinin is an antifibrinolytic agent, which is widely applied during open-heart operations to reduce the postoperative bleeding [3,4]. The high-cost of high-doses of aprotinin used in cardiac surgery and some lack of definite usefulness in extracardiac operations make the thoracic surgeons hesitant to use it. In fact, its use in extracardiac thoracic operations has so far only been tested in a few studies [5-7]. It has been established that the perioperative blood loss was significant reduced after infusion of 2 × 10^6 ^units of aprotinin [5,6]. The aim of our study is to evaluate if ultra-low dose of aprotinin (500.000 IU × 2), has any potential beneficial effect on blood losses and transfusion requirements as well as on the overall hospital outcome after major thoracic operations.

## Methods

### Patient population and study criteria

Fifty-nine patients (53 males and 6 females) mean age 58.03 ± 13.25 years that underwent major thoracic operations were included in this randomized and placebo-controlled study (table 1).

**Table 1:** Clinico-pathological parameters.

		**Group A**	**Group B**	**Total**
***Parameters***	Age	58.5 ± 9.8	57.5 ± 16.3	58.0 ± 13.2
	Gender(male/females)	27/3	26/3	53/6
	BMI*	25.2 ± 3.8	24.8 ± 3.9	25.0 ± 3.8

***Diagnosis***	Bronchiectasis	1	1	2
	Bullous disease	3	3	6
	Diaphragmatic tumors	0	1	1
	Empyema	4	4	8
	Esophageal achalasia	1	0	1
	Lung carcinoma	18	17	35
	Pulmonary metastases	2	2	4
	Thymoma	1	1	2
	
	**Total**	**30**	**29**	**59**

***Co-morbitidy***	COPD	7	6	13
	Diabetes Mellitus	4	6	10
	Hypertension	7	9	16
	Serum Crea: 1.5–2.0 mg/dl	1	1	2
	Past history of cancer	2	1	3
	Coronary artery disease	3	3	6
	Extracardiac arteriopathy	1	1	2
	WBC's >15,000 mm/cm^3^	1	0	1

***Pathology***	Primary lung carcinoma	18	17	35
	Other organ carcinoma	3	4	7
	Primary inflammatory disease	5	5	10
	Other	4	3	7
	
	**Total**	**30**	**29**	**59**

*BMI: Body Mass Index.

COPD = Chronic Obstructive Pulmonary Disease, WBC's = White Blood Count.

The preoperative exclusion criteria for enrollment in the study were: 1) known blood coagulation disorders, 2) known allergy to aprotinin (history of previous aprotinin infusion), 3) pre-operative coagulation defects expressed as PT or PTT > 1,5× control time or platelets count < 150 × 10^3^/cm^3^, 4) pre-operative treatment with intravenous heparin, coumarins, aspirin, or other anti-platelets factors and 5) pre-operative serum creatinine level greater than 2.0 mg/lt.

The intra-operative exclusion criteria were: 1) massive bleeding due to accidental injury to pulmonary artery or vein, 2) emergency re-operation for bleeding and 3) massive transfusion for any other reason.

Patients the morning before operation were randomly categorized by using relevant randomization tables, into placebo-group (Group A, n = 30) and aprotinin-treatment-group (Group B, n = 29). Only the anesthetist knew whether aprotinin was applied or not, thus, avoiding any possible influence on hemostasis and coagulation by the surgeons intraoperatively. The surgeons were informed about randomization after the patient has been transferred to the ward. The study was approved by the hospital ethics committee on human research and every patient included signed the appropriate consent form.

### Surgical procedures

All procedures were performed by the same surgical team. The majority of the procedures were performed through a lateral thoracotomy at the 5^th ^intercostal space most often for lung resection, except two medium sternotomies (table 2). After pulmonary excision and meticulous hemostasis, 1 or 2 chest-tubes 32 Fr were inserted, one to the top (anteriorly) and the other to the base (posteriorly). Negative pressure suction of 20 cm H_2_0 was utilized electively and only if residual pneumothorax was present. Drains were removed when no air leak was evident and when the last 12 hour drainage-fluid was less than 100 ml with the lung expanded. All patients received peri-operatively subcutaneous prophylactic administration of low molecular weight of tinzaparine sodium (Innohep^®^, Leo, Denmark) 3.500 Anti-Xa IU/0.35 ML 2–4 hours preoperatively and 1 per day up to the day of discharge.

**Table 2:** Types of performed operations.

		**Group A**	**Group B**	**Total**
***Operation***	Wedge resection	2	2	4
	Lobectomy	15	13	28
	Bilobectomy	1	1	2
	Pneumonectomy	3	4	7
	Maximal thymectomy	1	1	2
	Resection of diaphragmatic tumor	0	1	1
	Decortication	4	4	8
	Bullectomy	3	3	6
	Esophageal myotomy	1	0	1

***Total***		**30**	**29**	**59**

### Aprotinin dosage

The patients in the aprotinin-group (Group B) received immediately after intubation a test-dose of 1 ml solution of aprotinin (Trasylol^®^, Bayer AG, Leverkusen, Germany) followed by 500.000 IU of aprotinin intravenously in 50 ml of prepared manufactured solution administered over a period of 15 minutes. The same dose was given to the patient after thoracotomy closure. In contrast patients belonging to the placebo-group (Group A) received 50 ml of normal saline 0.9% solution before and after thoracotomy. No other haemostatic regiment was used. The intraoperative blood losses were properly recorded and the patient's vital signs were monitored. Intraoperatively or while staying in the recovery area patients were transfused with packed red blood cells (PRBC's) if Hb < 9 g/dl, while postoperatively, transfusion was performed to those with Hb < 8.5 g/dl. Fresh frozen plasma (FFP) was given on a clinical basis if no obvious clots were observed at the end of the procedure, or otherwise required because of clotting screen disturbances.

### Postoperative Care

The majority of patients (n = 50) underwent high thoracic catheter epidural analgesia for the first 3 postoperative days. The remaining received analgesia with intramuscularly administered pethidine chloride. As a routine, only 3 doses of perioperative antibiotic prophylaxis with ticarcillin & clavulanic acid 5.2 gr (Timentin^® ^Injections, GlaxoSmithKline, UK) commencing prior to induction to anesthesia, except of 6 patients in group A and 5 in group B with inflammatory pathology, who received appropriate antibiotic chemotherapy according to cultures. The amount of postoperative drainage volumes were recorded, as well as the hemoglobin level, the platelet number, the WBC, the clotting profile and the creatinine level, on the post-operative days 1^st^, 3^rd^, 5^th ^and the 7^th ^(for those discharged after the 7^th ^PO day) or at the day of discharge when earlier.

### Statistical analysis

All parametric values were expressed as mean measurement ± Standard Deviation (SD) or as a number. At the tables, the standard error of mean referred as SEM, minimum as Min and maximum as Max. Comparisons of continuous normally distributed data among the groups were performed by Student's t-test, p values < 0.05 were considered significant. When Shapiro-Wilk test of normality revealed that data did not conform to a normal distribution, a non-parametric test was used. To evaluate the groups in a nonparametric way, we used chi-square test (nominal data) or the Mann-Whitney *U *test (ordinal data) and when the probability was p < 0.05, we considered the groups dissimilar. Analyses were performed using SPSS for Windows, release 11.0.1 (SPSS Inc, Chicago, IL).

## Results

The 2 groups were similar in terms of age, gender, diagnosis, pathology, co-morbidity and operation performed (Tables 1, 2). There was no mortality and there was no significant difference in morbidity. Major complications included lung atelectasis due to sputum retention (n = 1, Group A), acute respiratory failure requiring mechanical ventilation (n = 1, Group B), prolonged air leak lasting more than 10 days (n = 3, two patients for group A and one for group B) and one case of atrial fibrillation (group A). There was no reoperation for bleeding. The mean values of preoperative laboratory parameters are showing in detail at the table 3. There was no statistical significant difference between the two groups for all of the parameters.

**Table 3:** Preoperative Laboratory data

***Parameters***	***Group A^†^***	***Group B^†^***	***Total^†^***
***Ht***	40.69 ± 0.80	41.27 ± 0.65	40.98 ± 0.51
***WBC***	8,821.00 ± 498.54	8,170.00 ± 400.25	8,501.02 ± 320.98
***PLT***	260,200 ± 15,850	302,414 ± 24,537	280,949 ± 14,643
***PT***	13.39 ± 0.41	12.57 ± 0.24	12.99 ± 0.25
***PTT***	33.8 ± 0.90	33.83 ± 1.25	33.6 ± 0.76
***INR***	1.06 ± 0.04	1.01 ± 0.02	1.04 ± 0.02
***Fibrinogen***	496.97 ± 24.63	471.28 ± 26.55	484.34 ± 18.01
***D-dimers***	0.84 ± 0.18	0.84 ± 0.21	0.84 ± 0.14
***Urea***	38.13 ± 2.15	37.76 ± 3.03	37.95 ± 1.83
***Crea***	0.94 ± 0.04	0.98 ± 0.05	0.96 ± 0.03
***SGOT***	21.13 ± 1.60	27.52 ± 3.05	24.27 ± 1.74
***SGPT***	29.33 ± 8.22	37.93 ± 6.84	33.56 ± 5.35
***Total Protein***	7.27 ± 0.10	7.36 ± 0.14	7.31 ± 0.08
***Albumin***	4.00 ± 0.10	4.16 ± 0.13	4.07 ± 0.8
***Total Bil***	0.61 ± 0.09	0.63 ± 0.06	0.62 ± 0.05
***Direct Bil***	0.11 ± 0.02	0.12 ± 0.01	0.11 ± 0.01

†values are express in terms of Mean ± SEM (SEM denotes Standard Error of Mean).

Ht = Hematocrit, WBC = white blood count, PLT = Platelets, PT = Prothrombin time, PTT = Partial thromboplastin time, INR = international normalized ratio, Creat = Creatinine, SGOT = serum glutamic oxaloacetate transaminase, SGPT = serum glutamic pyruvic transaminase, Bil = Bilirubin

### Post-operative blood loss and transfusion requirements

The mean post-operative blood loss during the 1^st ^post-operative day was 764.3 ± 213.9 ml in the placebo-group and 412.6 ± 199.2 ml in the aprotinin-group (p = 0.001). For the 2^nd ^day the blood loss was 455.0 ± 274.6 ml in the placebo group and 248.3 ± 178.5 ml in the aprotinin-group (p = 0.001) (Table 4). The intraoperative transfusion requirements in terms of PRBC's were similar in both groups: 0.17 vs 0.17 PRBC's (p = 0.967). Similarly the intraoperative need for fresh frozen plasma (FFP) transfusion was almost the same in both groups 0.20 vs 0.21 units (p = 0.330) Postoperatively after the patient left the recovery room up to the time of discharge, the need for PRBC's transfusion was higher in the placebo-group, but the difference was not significant: 0.03 vs 0.00 (p = 0.970). The post-operative transfusion of FFP was significant higher in the placebo group: 0.87 Units vs 0.21 Units (p = 0.035) (Table 5).

**Table 4:** Post-operative blood loss.

***Parameters***	***Value***	***Group A***	***Group B***	***p***
***Day 1 Postoperative Thoracic Drainage (ml)***	Mean	764.3	412.6	0.000
	Min	350.0	150.0	
	Max	1230.0	900.0	
	SEM	39.1	37.0	
	SD	213.9	199.2	

***Day 1 Postoperative Thoracic Drainage (ml)***	Mean	455.0	248.3	0.001
	Min	.0	.0	
	Max	1100.0	700.0	
	SEM	50.1	33.2	
	SD	274.6	178.5	

Post operative blood loss. Mean post-operative blood loss at 1st and 2nd postoperative day. SD = Standard deviation, Min = minimal, Max = maximal, SEM = Standard Error of Mean.

**Table 5:** Transfusion requirements

***Parameters***	***Value***	***Group A***	***Group B***	***p***
***Intraoperative PRBC's Units***	Mean	0.17	0.17	0.967
	SD	0.531	0.539	
	Min	0	0	
	Max	2	2	

***Postoperative PRBC's Units***	Mean	0.03	0.00	0.970
	SD	0.183	0.000	
	Min	0	0	
	Max	1	0	

***Intraoperative FFP's Units***	Mean	0.20	0.21	0.330
	SD	0.761	0.620	
	Min	0	0	
	Max	3	2	

***Postoperative FFP's Units***	Mean	0.87	0.21	0.035
	SD	1.525	0.620	
	Min	0	0	
	Max	4	2	

Intraoperative and post-operative transfusion requirements. *PRBC's = Packed Red Blood Cells. FFP's = Fresh Frozen Plasma*.

### Postoperative Hematocrit and white blood cell count

The values of hematocrit at the 1^st ^and 2^nd ^postoperative day and at discharge were all significantly higher in the aprotinin-group: 35.9 vs 33.0, 34.5 vs 32.1 and 35.0 vs 32.0 (p = 0.07, 0.026, and 0.035 respectively) (Table 6). There was no difference between the two groups in the preoperative white blood count (WBC) (p = 0.313). However, there was a considerable elevation in both groups during the two first postoperative days, more marked in the group A, but not statistically significant. At the 7^th ^day (for those discharged after the 7^th ^day) the WBC was significantly preserved for group B (8614.3 ± 2328.9 vs 13168.2 ± 3497.4) (p = 0.001) (Table 7).

**Table 6:** Hematocrit values.

***Parameters***	***Value***	***Group A***	***Group B***	***p***
***Preoperative Hematocrit***	Mean	40.7	41.3	0.573
	Min	28.2	35.5	
	Max	48.0	50.2	
	SEM	0.8	0.6	
	SD	4.4	3.5	

***Day 1 Postoperative Hematocrit***	Mean	33.0	35.9	0.007
	Min	25.7	28.0	
	Max	42.8	44.9	
	SEM	0.7	0.7	
	SD	4.1	3.9	

***Day 2 Postoperative Hematocrit***	Mean	32.1	34.5	0.026
	Min	24.2	25.7	
	Max	39.0	41.8	
	SEM	0.7	0.7	
	SD	4.1	3.9	

***Discharge Hematocrit***	Mean	32.0	35.0	0.035
	Min	26.7	24.3	
	Max	39.0	43.5	
	SEM	1.3	1.3	
	SD	4.1	4.7	

Hematocrit values at the 1st, 2nd postoperative day and at discharge.

**Table 7:** Perioperative White Blood Count values.

***Parameters***	***Value***	***Group A***	***Group B***	***p***
***Preoperative WBC***	Mean	8,821.0	8,170.0	0.313
	SD	2,730.6	2,155.4	
	Min	4,970.0	3,900.0	
	Max	16,300.0	13,710.0	

***Day 1 Postoperative WBC***	Mean	11,564.7	10,470.0	0.296
	SD	4,822.2	2,869.7	
	Min	5,840.0	5,820.0	
	Max	28,700.0	16,890.0	

***Day 2 Postoperative WBC***	Mean	10,644.3	9,610.7	0.223
	SD	3,494.1	2,832.5	
	Min	5,550.0	5,000.0	
	Max	17,710.0	16,490.0	

***Day 7 Postoperative WBC***	Mean	13,168.2	8,614.3	0.001
	SD	3,497.4	2,328.9	
	Min	7,600.0	5,260.0	
	Max	18,350.0	14,400.0	

White blood count values at 1st, 2nd and at 7th postoperative day (for those patients discharged after the 7th PO day (WBC = White Blood Count).

### Operation time and Postoperative hospital stay

The operation-time was lower in the aprotinin-group but the difference was not significant: 84.66 ± 35.23 min vs 101.17 ± 52.45 min (p = 0.161). However, the postoperative hospital-stay was longer in the placebo-group, but without reaching statistical significance (7.2 ± 3.61 days vs 5.83 ± 1.65 days) (p = 0.064) (Table 8).

**Table 8:** Operation time and postoperative stay.

***Parameters***	***Value***	***Group A***	***Group B***	***p***
***Operation Time (min)***	Mean	101.17	84.66	0.161
	Min	40.00	40.00	
	Max	270.00	180.00	
	SEM	9.58	6.54	
	SD	52.45	35.25	

***Postoperative Hospital stay (days)***	Mean	7.20	5.83	0.064
	Min	4.00	3.00	
	Max	21.00	10.00	
	SEM	0.66	0.31	
	SD	3.61	1.65	

The operation-time and postoperative stay were lower in the aprotinin-group reaching statistical significance.

## Discussion

Aprotinin is generally regarded as an effective hemostatic agent that preserves platelet function thus leading to postoperative blood loss reduction.

Aprotinin was discovered in 1930, but it was clinically applied much later, in the early 1960s for the treatment of acute pancreatitis. It's a protease inhibitor that exerts its effect at different sites in the inflammatory cascade. In particular, aprotinin inhibits the activity of kallikrein, which consequently decreases the activation of complement and bradykinin [8]. Due to this action aprotinin is widely applied in cardiac operations using cardiopulmonary bypass [3,4]. During these operations, the extracorporeal circulation activates kallikrein, which liberates factor XII, complement, plasmin and bradykinin. All these increase the inflammatory injury, the capillary permeability and coagulation abnormalities and represent sub-bases of the systemic inflammatory response syndrome (SIRS) [3,8].

In a recent systematic review and meta-analysis of randomized clinical trials, Sedrakyan et al. [9] evaluated the effect of aprotinin on 3878 patients undergoing coronary surgery and found that not only it reduces the transfusion needs, but also it is associated with no post-operative complications and therefore its use could be expanded into coronary surgery with no reluctance. In fact there was a tendency toward reduction of atrial fibrillation occurrence in those who received aprotinin. Dignan et al. using an ultra-low dose of aprotinin (500.000 × 2 KIU) reported a beneficial effect and supported its routine use in coronary surgery [10].

Since the introduction of aprotinin in clinical practice, it has also been used in other types of surgery where beneficial effects were expected. There have been some reports which have demonstrated that the use of aprotinin is effective in reducing the need of blood transfusion after liver transplantation [11] and major orthopedic surgery [12].

In thoracic surgery and particularly in high risk patients where severe post-operative bleeding is often anticipated, use of aprotinin has been similarly tested in clinical trials. Bedirhan et al. [6], in a randomized trial using 10^6 ^kallikrein inhibitory units (KIU) at the beginning and followed by an infusion of the same dose during chest closure, showed that transfusion requirements and postoperative bleeding was reduced. Kyriss et al. [5], in a placebo-controlled randomized phase IV study, evaluated the effect of aprotinin in thoracic surgical operations. They initially infused 2 × 10^6 ^KIU followed by 5 × 10^5 ^KIU/hr during surgery. They similarly reported a reduction in both perioperative blood loss and blood transfusion.

Major thoracic surgery is associated with a considerable risk of bleeding. For pulmonary excisions this risk is about 4.6% [2] and re-exploration thoracotomy for hemorrhage is required in 3.7% [13]. Furthermore, SIRS may arise as a result not only following open-heart operations but also after a major trauma or a major operation [14]. The beneficial effect of aprotinin, namely inhibition of contact activation and fibrinolysis while preserving platelet function has not been tested so far in clinical trials using rather lower doses as in the present study. Regarding post-operative blood loss, we demonstrated that this was significantly higher for the placebo-group: 764.3 ± 213.9 ml vs 412.6 ± 199.2 ml and 455.0 ± 274.6 ml vs 284.3 ± 178 ml for the first end second postoperative days comparing to the aprotinin-group (p = 0,001) (Table 4). This higher blood loss may explain the significant difference in the hematocrit level in favor of group B during postoperative period (Table 6). We have also showed that there was no significant difference between the two groups concerning the blood transfusion requirements, either intraoperatively or postoperatively (Table 5). In our opinion, these differences are attributed to three reasons: a) the patients with perioperative massive surgical bleeding were excluded from the study, b) our postoperative transfusion criteria were very strict and we avoided transfusing patients with hemoglobin higher of 8.5 g/dl. This is very important guideline for transfusing patients in thoracic surgery as it has been previously reported by our group [15]. And c) we are not reluctant in transfusing FFP's if there is a bleeding tendency and severe oozing with no clot formation, even on a clinical ground. As a result, the amount of post operative transfused FFP's was significant higher in the placebo-group (0.87 Units vs 0.21 Units for the aprotinin-group), (p = 0.035) (Table 5). This relation itself represents an indirect evidence of prevailing hemorrhagic diathesis of patients in placebo-group.

We also observed postoperative preservation on the number of leukocytes in the aprotinin-group in contrast to the placebo-group. This finding is attributed to the anti-inflammatory action of aprotinin. There is evidence that aprotinin modifies the inflammatory response through an inhibition of neutrophil activation [16]. It has already been confirmed, the reduced ability of leukocytes to attach along the vessel wall, to firm adhesion and to transmigrate into the extravascular space under the influence of aprotinin [17,18]. Furthermore, Asimakopoulos et al. showed experimentally that infusion of aprotinin inhibits significantly the leukocyte extravasation [14].

An unexpected finding from our study was the difference in operating time between the groups. In the placebo-group the average operating time was 101.17 ± 52.5 min, while in the aprotinin-group was only 84.6 ± 35.23 min. Although it was not statistically significant, there was tendency to be so (Table 8). This difference reflected the additional time demanded for appropriate intraoperative hemostasis.

Another remarkable result of our study was the lower postoperative hospital stay observed in the aprotinin-group (7.2 ± 3.6 days for the placebo-group vs. 5.83 ± 1.65 days for the aprotinin-group). This difference, which has an obvious tendency to be significant in favor of aprotinin usage, (p = 0,064) (Table 8) was related to the lower incidence of bleeding in the aprotinin-group on the one hand, and to the anti-inflammatory activity of aprotinin as already mentioned, including the preservation of the WBC during postoperative course.

There are two main disadvantages of aprotinin, namely, the possibility of eliciting allergic reactions and the high cost. The allergic adverse effects are rare, less than 0.1–0.2% [3,19]. In our study, no allergic reactions were observed. In terms of cost, the usual dose of aprotinin in extracardiac operations ranges between 2 × 10^6 ^to 2.5 × 10^6 ^Units with an average cost between 48 to 60 € (Patras, Greece). In our study, using a total of 10^6 ^KIU, the total cost per patient per operation was 24 €, which we believe it is definitely cost beneficial. Nevertheless, any cost of aprotinin is compensated by that of potential transfusion of blood and its products, and possibly by that of a longer operation time and hospital stay [20].

## Conclusion

In summary, the ultra-low dose of aprotinin tested in this study was associated with a reduction of total blood losses and blood product requirements as well as with lower intraoperative time and overall hospital stay. We therefore consider the use of aprotinin safe and recommend its use in thoracic surgery and in particular, when potential perioperative bleeding is anticipated.

## Competing interests

The author(s) declare that they have no competing interests.

## Authors' contributions

All authors: 1) have made substantial contributions to conception and design, or acquisition of data, or analysis and interpretation of data; 2) have been involved in drafting the manuscript or revising it critically for important intellectual content; and 3) have given final approval of the version to be published.
